# Vitamin D and metabolic disturbances in polycystic ovary syndrome (PCOS): A cross-sectional study

**DOI:** 10.1371/journal.pone.0204748

**Published:** 2018-12-04

**Authors:** Y. H. M. Krul-Poel, P. P. Koenders, R. P. Steegers-Theunissen, E. ten Boekel, M. M. ter Wee, Y. Louwers, P. Lips, J. S. E. Laven, S. Simsek

**Affiliations:** 1 Department of Internal Medicine/Endocrinology, VU University Medical Center, Amsterdam, The Netherlands; 2 Department of Internal Medicine, Medical Center Alkmaar, Alkmaar, The Netherlands; 3 Department of Pediatrics, Division of Neonatology, Erasmus MC, University Medical Center, Rotterdam, The Netherlands; 4 Department of Obstetrics and Gynecology, Erasmus MC, University Medical Center, Rotterdam, The Netherlands; 5 Department of Clinical Chemistry, Hematology & Immunology, Medical Center Alkmaar, Alkmaar, The Netherlands; 6 Department of Epidemiology and Biostatistics, VU University Medical Center, Amsterdam, the Netherlands; 7 Division of Reproductive Medicine, Department of Obstetrics, Gynecology, Erasmus Medical Center Rotterdam, The Netherlands; University of Tennessee Health Science Center, UNITED STATES

## Abstract

**Objective:**

To compare vitamin D status in women with PCOS versus fertile women and subsequently evaluate the association between vitamin D status and metabolic disturbances in PCOS women.

**Methods:**

We conducted a cross-sectional comparison study of 639 women with PCOS and 449 fertile women. Serum 25-hydroxyvitamin D (25(OH)D) was stratified into a severe deficient (< 25 nmol/l), insufficient (25–50 nmol/l), moderate (50–75 nmol/l) and adequate (> 75 nmol/l) status. The main outcome measures were the difference in vitamin D status between PCOS and fertile women, and the association between serum 25(OH)D and metabolic disturbances in PCOS women only.

**Results:**

Serum 25(OH)D was significantly lower in PCOS women compared to fertile controls (mean 25(OH)D of 49.0 nmol/l *versus* 64.5 nmol/l). An adjusted significant difference was seen between serum 25(OH)D and homeostasis model assessment (HOMA-IR) (β = 0.76; 95% CI: 0.63–0.91; p < 0.01), HDL-cholesterol (β = 0.20; 95% CI: 0.05–0.60, p < 0.01) and apolipoprotein A1 (β = 26.2; 95% CI: 7.5–45.0, p < 0.01) between the highest vitamin D group compared to the lowest vitamin D group.

**Conclusions:**

This study demonstrates that women with PCOS have a significantly lower serum 25(OH)D compared to fertile controls. A compromised vitamin D status in PCOS women is associated with a higher HOMA-IR and an unfavourable lipid profile. Large randomized controlled trials are necessary to explore the causality of this linkage.

## Introduction

Polycystic ovary syndrome (PCOS) is the most common endocrinopathy in women of reproductive age, with a prevalence up to 10% depending on which diagnostic criteria are used [1]. It is characterized by ovulatory dysfunction, hyperandrogenism and/or polycystic ovarian morphology [2]. Metabolic disturbances are present in a majority of the women suffering from PCOS, i.e. 30–40% have impaired glucose tolerance and insulin resistance with compensatory hyperinsulinemia, and as many as 10% will develop type 2 diabetes mellitus during their fourth decade [3]. Adipose tissue dysfunction has been implicated as a contributor to insulin resistance in women with PCOS. However, a substantial number of lean women affected by PCOS have insulin resistance as well, independent of obesity [4,5].

Vitamin D deficiency has been proposed as the possible missing link between insulin resistance and PCOS [6]. Vitamin D is a fat-soluble vitamin that is synthesized endogenously through sunlight-induced photochemical conversion of cholesterol to 7-dehydrocholesterol in the skin or obtained from the diet. Subsequently vitamin D undergoes a hydroxylation twice, first vitamin D is transported to the liver where it is rapidly hydroxylated by 25-hydroxylase into 25-hydroxyvitamin D (25(OH)D). The second hydroxylation occurs in the kidney and is catalyzed by 1 alpha-hydroxylase to form 1,25-dihydroxyvitamin D (1,25(OH)_2_D), the active metabolite of vitamin D. Circulating 1,25(OH)_2_D binds to vitamin D receptors (VDR) to initiate its effect. Serum 25(OH)D is the major circulating form of vitamin D and is used as the main indicator of vitamin D status. Its half-life is 2–3 weeks compared to only 4–6 hours for 1,25(OH)_2_D [7]. Since many years, a role for vitamin D has been suggested outside the calcium and bone homeostasis, due to the identification of the VDR, and the enzyme 1 alpha-hydroxylase in many more tissues, including the pancreatic beta-cells, immune cells [8] and reproductive organs in both genders [6]. Moreover, this assumption is supported by the finding that the active vitamin D-vitamin D receptor complex regulates over 300 genes, including genes that are important for glucose and lipid metabolism as well gonadal function [9].

Observational studies have demonstrated a link between vitamin D deficiency and the onset of and progression of type 2 diabetes [10–13]. Furthermore, low vitamin D status is associated with future macrovascular events in patients with type 2 diabetes mellitus [14]. This association may be the result of the link between vitamin D status and renin-angiotensin system [15], endothelial function [16], blood pressure [17] or chronic inflammation [18]. Women with PCOS seem to have a higher risk for vitamin D deficiency as well [19]. Associations of vitamin D status and metabolic disturbances have been investigated in a large number of studies, which are summarized in a systematic review. However, due to the heterogeneity of the studies, small sample sizes, and small number of studies no firm conclusion could be drawn [20]. In addition, the association between vitamin D and insulin resistance has been studied thoroughly in patients with diabetes, resulting in convincing evidence from observational studies that vitamin D deficiency is inversely related to the degree of insulin resistance [21].

So far, it is not clear whether vitamin D deficiency is a risk factor for PCOS, and whether vitamin D status is associated with metabolic disturbances in PCOS women. Therefore, we performed a cross-sectional comparison study to examine vitamin D status in PCOS women and fertile women. We also did an analysis in this large PCOS cohort to extend the current knowledge about the association between vitamin D and metabolic disturbances in PCOS women.

## Materials and methods

### Subjects

This retrospective comparison study included 639 PCOS women from the Rotterdam PCOS cohort and 449 fertile women from the HAVEN cohort, which are further mentioned throughout the manuscript as control women. PCOS women who were screened for anovulatory infertility at the outpatient clinic of the Erasmus Medical Center Rotterdam, and subsequently diagnosed with PCOS according to the Rotterdam criteria were eligible for inclusion in this study. Trained professionals performed the screening procedure according to a standardized protocol which has been previously described in greater detail [22]. First, a thorough general medical, reproductive and family history was taken, including self-reported ethnicity. Second, anthropometric measurements were performed, including height, weight, body mass index (BMI), waist circumference measured midway between the arcus costae and anterior superior iliac spine, hip circumference measured at the level of the anterior superior iliac spine, systolic and diastolic blood pressure, and the level of hirsutism measured with the use of the Ferriman-Gallwey (FG) score. Subsequently, a systematic transvaginal ultrasonography was performed to assess ovarian volume, endometrial thickness, and the total number of antral follicles measuring 2–10 mm. Finally, an extensive metabolic and endocrine profile was assessed.

PCOS was diagnosed according to the Rotterdam criteria; i.e., requiring the presence of at least two out of the three following criteria: ovulatory dysfunction resulting in oligomenorrhea (mean bleeding interval 35–182 days in last six menstrual bleeds) and/or amenorrhea (absence of menstrual bleeding for >182 days), hyperandrogenism and/or hirsutism, and the presence of polycystic ovarian morphology (PCOM) [23]. Clinical and biochemical hyperandrogenism was defined as an FG score >8, and/or a free androgen index (FAI: [(T/SHBG) x 100] >4.5) [24]. Women were excluded if the blood draw was not performed in a fasting state.

Control women were recruited at child health centers in the same geographic area as the study population. This group consisted of a random group of mothers who had a spontaneous and uneventful pregnancy and delivered a healthy child without congenital malformations. They were all assessed, at 15–17 months after delivery at the hospital using a standardized study protocol. These women have been described previously [24,25]. Information on general health and cycle history was gathered by questionnaire. This study was approved by the Central Committee on Research Involving Human Subjects (the Hague, The Netherlands) and the institutional review board at the Erasmus Medical Center. Informed consent was obtained from all participants.

#### Assessment of metabolic profile and vitamin D

Regarding the cardiovascular profile, information was collected on BMI and systolic and diastolic blood pressure in both PCOS and comparison women. In PCOS women, waist circumference, fasting glucose, insulin and lipid profile (i.e. total cholesterol, triglycerides, high-density lipoprotein cholesterol and low-density lipoprotein cholesterol) were additionally measured. Insulin resistance was assessed using the homeostasis model assessment (HOMA-IR: [fasting glucose (mmol/L) x fasting insulin [mU/L]]/22.5). Metabolic syndrome was defined according the National Cholesterol Education Program (NCEP) ADP III criteria [26].

Venous blood samples were drawn at examination and stored at −80°C after centrifugation at 3000 rpm for 10 min at 20°C. Serum 25(OH)D was measured using the liquid chromatography tandem-mass spectrometry (LC-MS/MS) in November 2013 (PCOS women) and September 2015 (control women). Liquid-liquid extraction (hexane) of the samples and analysis, carried out with a Waters ACQUITY UPLC system couple to a Waters Xevo TQ mass spectrometer, were performed as described elsewhere [27]. Intra-assay coefficients of variation (CV) was < 6% and intra-assay CV was < 9% at the concentration of 16 and 80 nmol/L, respectively. The measurement of serum 25(OH)D was carried out by the central chemical laboratory of the Medical Center Alkmaar, the Netherlands. This laboratory is a (ISO-15189) certified laboratory.

Endocrine evaluation included serum levels of gonadotropic hormones (LH, FSH) and estradiol (E_2_), testosterone, dehydroepiandrosterone sulfate (DHEAS), fasting glucose and insulin. Hormone assays have been described in detail elsewhere [28]. Insulin was measured by immunoradiometric assay. Testosterone, E_2_ and DHEAS were determined by radioimmunometric assays (RIAs). Intra-assay and inter-assay CV were less than 3% and less than 5% for testosterone and less than 5% and less than 7% for E2 respectively.

### Statistical analyses

Data are presented as means ± standard deviation if normally distributed, and as median and interquartile range in case of a skewed distribution. For the main analyses we compared PCOS women with fertile controls regarding vitamin D status. We used a linear regression analysis with serum 25(OH)D as dependent variable and PCOS versus control as independent variable. We adjusted for BMI, age, blood pressure, season of vitamin D measurement, and ethnicity as potential confounders or effect modifiers. Serum 25(OH)D was log transformed due to a non-gaussian distribution. The exponentiated beta-coefficients are reported as results of the regression analyses.

Sub-analyses were performed to assess the association between vitamin D status and metabolic outcome parameters in women with PCOS, by applying multivariable linear regression analyses. As the relationship between serum 25(OH)D and the outcome measures was not linear, serum 25(OH)D was divided in four groups by the widely used cut-off values of vitamin D: 1) serum 25(OH)D: < 25 nmol/l, 2) serum 25(OH)D: 25–50 nmol/l, 3) serum 25(OH)D: 51–75 nmol/l, and 4) serum 25(OH)D: > 75 nmol/l [20]. The vitamin D group with a serum 25(OH)D > 75 nmol/l was used as reference value. Regarding the lipid profile the highest vitamin D group (> 75 nmol/l) was compared to the lowest vitamin D group (< 25 nmol/l). We adjusted for confounding variables which are known to influence the association between vitamin D level and cardiovascular outcomes (e.g. BMI, lipid profile, ethnicity etc.). A p-value < 0.05 was considered statistically significant. All data were analysed using the Statistical Package of the Social Sciences (SPSS software, version 22.0; SPSS Inc., Chicago, IL).

## Results

### Vitamin D status in PCOS and control women

A total of 639 PCOS women and 449 fertile control women were included in the analyses. Demographic, anthropometric and clinical characteristics are presented in Table 1. The mean age was 34 ± 5 and 32 ± 5 years, with a median BMI of 25.2 (22.0–30.4 kg/m^2^) and 24.0 (22.0–27.0 kg/m^2^) in PCOS and control women, respectively. Overall median serum 25(OH)D was 49.0 (27.1–74.1 nmol/l) in PCOS versus 64.5 (39.2–85.7) in the control women. A severe vitamin D deficiency was present in 136 (21%) women out of 639 PCOS women, 190 (30%) women had a serum 25(OH)D between 25.1 and 50.0 nmol/l, 165 (26%) women had a serum 25(OH)D between 50.1 and 75.0 nmol/l, and 148 (23%) women had a serum 25(OH)D > 75 nmol/l. In the control group 49 (11%) women had a serum 25(OH)D ≤ 25 nmol/l, 106 (24%) women between 25.1 and 50.0 nmol/l, 131 (29%) women between 50.1 and 75.0 nmol/l, and 163 (36%) women had a serum 25(OH)D > 75 nmol/l. Linear regression analysis showed a lower serum 25(OH)D for the PCOS women compared to control women (β = 0.78; 95% CI: 0.72–0.84, p < 0.01) (Table 2). Correcting this analysis for age, BMI, blood pressure, ethnicity and season the difference remained significant (β = 0.93; 95% CI: 0.87–0.99, p = 0.03).

**Table 1:** Baseline characteristics of PCOS and fertile control women.

	PCOS	Control	p-value
N	639	449	
Age (y)	34 ± 5	32 ± 5	< 0.01
Body Mass Index (kg/m^2^)	25.2 (22.0–30.4)	24.0 (22.0–27.0)	< 0.01
Ethnicity (%)			
Caucasian	385 (60)	382 (85)	< 0.01
Non-Caucasian	254 (40)	67 (15)	
Season of blood collection			
Winter	145 (23)	114 (25)	0.30
Spring	114 (18)	142 (32)	< 0.01
Summer	207 (32)	93 (21)	< 0.01
Autumn	173 (27)	200 (22)	0.07
BP systolic (mmHg)	118 ± 13	114 ± 11	< 0.01
BP diastolic (mmHg)	77 ± 11	73 ± 9	< 0.01
Serum 25(OH)D (nmol/l)	49.0 (27.1–74.1)	64.5 (39.2–85.7)	< 0.01
Storage of samples (years)	6	10	< 0.01

25(OH)D, 25-hydroxy vitamin D; BP, blood pressure.

**Table 2:** Linear regression analysis of serum 25(OH)D* in PCOS versus fertile controls.

	B (95% CI)	p
**Model 1:** *crude analysis*	0.78 (0.72–0.84)	< 0.01
**Model 2:** *Model 1 + age*, *BMI and systolic blood pressure*	0.79 (0.73–0.86)	< 0.01
**Model 3:** *Model 2 + season and ethnicity*	0.93 (0.87–0.99)	0.03

* serum 25(OH)D was natural log transformed

25(OH)D, 25-hydroxy vitamin D; BMI, body mass index

### PCOS women

Demographic, anthropometric and clinical characteristics of all PCOS women, stratified by vitamin D group are presented in Table 3. The majority (60%) of included women were of Caucasian ethnicity. The distribution of the various ethnicities differed significantly between women with a severe vitamin D deficiency compared to women with a sufficient vitamin D status, with 92% Caucasian women in the highest vitamin D group versus 14% of the women in the lowest vitamin D group. An expected difference was seen in season of measurement between the vitamin D groups, with the highest percentage of blood samples taken in the summer presenting in the highest vitamin D group and the lowest percentage of blood samples taken in the summer in the lowest vitamin D group (48% versus 19%, respectively).

**Table 3:** Baseline demographic and clinical characteristics.

		Vitamin D groups (serum 25(OH)D)			
	All	*< 25*.*0 nmol/l*	*25*.*0–50*.*0 nmol/l*	*50*.*1–75*.*0 nmol/l*	*> 75*.*0 nmol/l*
N	639	136	190	165	148
Age (y)	34 ± 5	32 ± 5	33 ± 6	34 ± 6	35 ± 5
Ethnicity (%)					
Caucasian	385 (60)	19 (14)	101 (53)	129 (78)	136 (92)
Non-Caucasian	254 (40)	117 (86)	89 (47)	36 (22)	12 (8)
Season of blood collection					
Winter	145 (23)	40 (29)	52 (27)	33 (20)	20 (14)
Spring	114 (18)	31 (23)	35 (19)	31 (19)	17 (11)
Summer	207 (32)	25 (19)	52 (27)	59 (36)	71 (48)
Autumn	173 (27)	40 (29)	51 (27)	42 (25)	40 (27)
Storage (y)	6 ± 1	6 ± 1	6 ± 1	6 ± 1	6 ± 1
Body Mass Index (kg/m^2^)	26.6 ± 6.0	29.1 ± 6	26.7 ± 6.1	26.4 ± 5.9	24.3 ± 5.3
Waist/hip ratio	0.8 ± 0.1	0.8 ± 0.1	0.8 ± 0.1	0.8 ± 0.1	0.8 ± 0.1
FG score	5 (1–8)	9 (2–13)	5 (1–7)	4 (0–5)	4 (0–6)
BP systolic (mmHg)	118 ± 13	119 ± 12	118 ± 13	118 ± 13	117 ± 13
BP diastolic (mmHg)	77 ± 11	78 ± 11	77 ± 10	77 ± 11	76 ± 9
Metabolic syndrome yes (%)	90 (15)	33 (24)	26 (14)	23 (14)	12 (8)
Serum 25(OH)D (nmol/l)	49.0 (27.1–74.1)	16.8 (12.9–21.4)	37.0 (30.0–43.4)	62.9 (56.3–69.9)	94.2 (81.7–102.4)
Fasting glucose (mmol/L)	4.33 ± 0.68	4.49 ± 0.73	4.37 ± 0.78	4.28 ± 0.57	4.20 ± 0.55
Fasting insulin (mU/L)	10.0 (4.6–12.4)	14.2 (6.2–18.2)	10.6 (5.3–12.8)	8.5 (4.6–10.9)	7.2 (3.5–9.4)
HOMA-IR	1.99 (0.85–2.46)	2.94 (1.13–3.66)	2.09 (0.95–2.48)	1.64 (0.85–2.13)	1.36 (0.66–1.72)
Total cholesterol (mmol/L)	5.1 ± 1.2	5.0 ± 1.3	5.1 ± 1.2	5.2 ± 1.3	5.2 ± 1.1
LDL-cholesterol (mmol/L)	3.6 ± 1.0	3.6 ± 1.0	3.6 ± 1.0	3.7 ± 1.1	3.7± 1.0
HDL-cholesterol (mmol/L)	1.4 ± 0.5	1.3 ± 0.4	1.4 ± 0.5	1.5 ± 0.5	1.6 ± 0.5
Triglycerides (mmol/L)	1.1± 0.7	1.2 ± 0.8	1.1 ± 0.6	1.2 ± 0.7	0.9 ± 0.4
TC/HDL ratio (mmol/L)	3.9 ± 1.4	4.3 ± 1.6	3.9 ± 1.3	3.8 ± 1.4	3.5 ± 1.3
Apo A1 (g/L)	213.3 ± 59.4	198.1 ± 47.6	215.4 ± 59.2	215.6 ± 67.8	221.9 ± 57.3
Apo B (g/L)	121.9 ± 37.9	126.5 ± 42.1	123.1 ± 37.0	120.8 ± 37.8	117.1 ± 34.6
Testosterone (nmol/L)	2.01 (1.40–2.50)	2.18 (1.50–2.70)	2.02 (1.30–2.60)	1.96 (1.40–2.40)	1.89 (1.20–2.35)
Free androgen index	6.5 (3.0–8.6)	9.3 (4.9–12.6)	6.7 (3.1–8.9)	5.6 (2.8–7.5)	4.6 (2.1–6.3)
Estradiol (pmol/L)	301 (176–361)	285 (181–361)	301 (179–356)	313 (175–361)	302 (168–394)
DHEAS (μmol/L)	5.36 ± 2.47	5.51 ± 2.89	5.36 ± 2.34	5.18 ± 2.35	5.42 ± 2.37
AMH (μg/L)	11.0 (6.0–14.2)	10.2 (5.8–12.7)	10.1 (5.6–13.6)	11.0 (6.0–14.9)	12.7 (6.8–15.1)

25(OH) D, 25-hydroxy vitamin D; AMH, antiMullerian hormone, APO, apolipoprotein; BP, blood pressure; DHEAS, dehydroepiandrosteron sulfate; FG, Ferriman–Gallwey; FSH, follicle stimulating hormone, HDL, high density lipoprotein; HOMA-IR, homeostatic model assessment–insulin resistance; LDL, low density lipoprotein; LH, luteinizing hormone, TC, total cholesterol; TG, triglycerides; TSH, thyroid stimulating hormone

### Anthropometric parameters

Median BMI of all PCOS women included was 25.2 (22.0–30.4 kg/m^2^). Overweight/obese women (BMI > 25 kg/m^2^) had a mean serum 25(OH)D 46.3 ± 27.9 nmol/l, which was significantly lower compared to women with a BMI < 25: serum 25(OH)D 59.7 ± 30.0 nmol/l (p < 0.01). A total of 94 (15%) women fulfilled the NCEP APT III criteria for metabolic syndrome, who had a significant lower serum 25(OH)D than women without metabolic syndrome (mean serum 25(OH)D 44.3 ± 27.4 nmol/l and 54.2 ± 29.9 nmol/l (p < 0.01), respectively).

### Glycemic parameters

**Fig 1:**
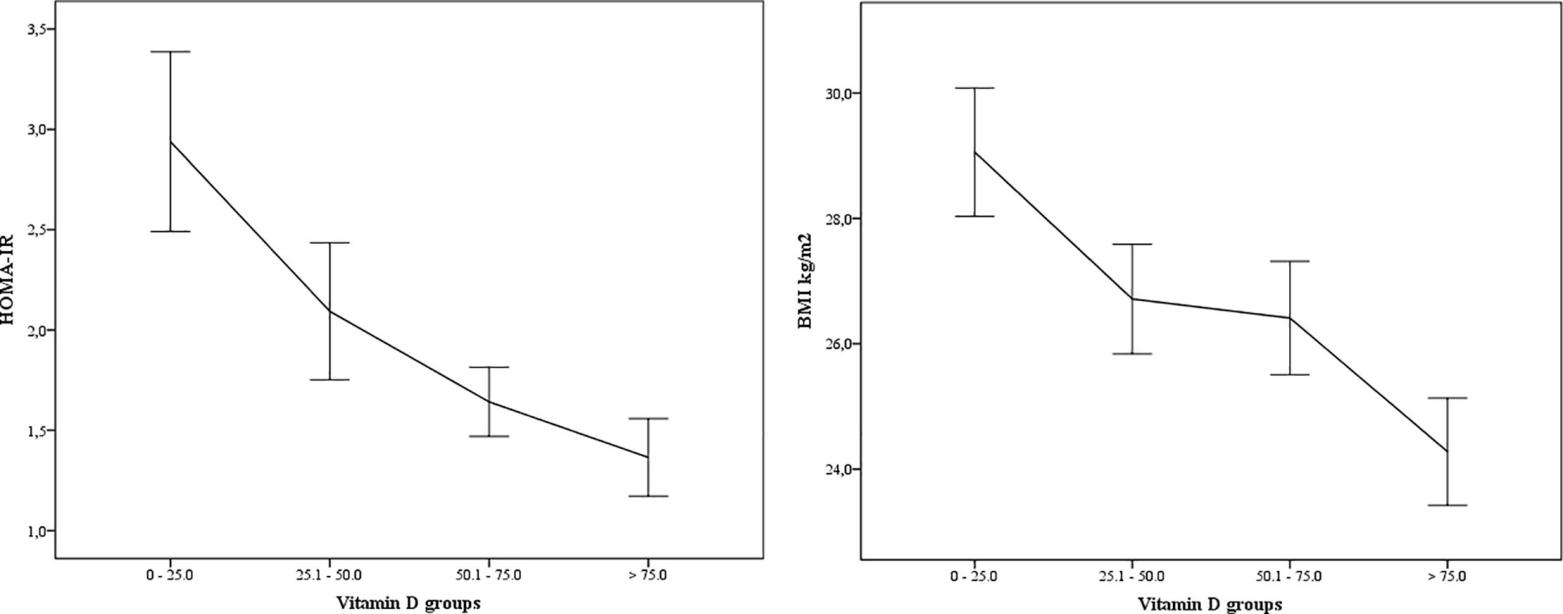
Association between serum 25(OH)D (nmol/l) groups and HOMA-IR and BMI.

Linear regression analysis correcting for BMI, ethnicity, waist to hip ratio, cholesterol/HDL ratio, and season resulted in a significant difference in HOMA-IR between the highest and lowest vitamin D groups. In the women with the lowest vitamin D level, HOMA-IR was on average 24% higher compared to the women with the highest vitamin D level (β = 0.76; 95% CI: 0.63–0.91; p < 0.01, Table 4) (Fig 1). Also, a significant difference was found between the middle vitamin D group with a serum 25(OH)D between 25.1 and 50.0 nmol/l and the highest vitamin D group (β = 0.83; 95% CI: 0.72–0.96, p = 0.01). No significant difference was found between the middle-high vitamin D group (serum 25(OH)D: 50.1–75.0 nmol/l) compared to the women with a serum 25(OH)D > 75 nmol/l regarding HOMA-IR. No significant difference between the vitamin D groups was seen in fasting glucose after correction for BMI, age, ethnicity and season (β = -0.18; 95% CI: -0.39–0.03, p = 0.09).

**Table 4:** Linear regression analysis of vitamin D groups* and HOMA-IR**.

	Exp(B) (95% CI)	p-value
**Model 1:** *crude analysis*		
1.Serum 25(OH)D ≤ 25.0 nmol/l	0.49 (0.41–0.58)	< 0.01
2.Serum 25(OH)D 25.1–50.0 nmol/l	0.68 (0.57–0.80)	< 0.01
3.Serum 25(OH)D 50.1–75.0 nmol/l	0.80 (0.67–0.94)	< 0.01
**Model 2:** *Model 1 + BMI*		
1.Serum 25(OH)D ≤ 25.0 nmol/l	0.70 (0.60–0.81)	< 0.01
2.Serum 25(OH)D 25.1–50.0 nmol/l	0.81 (0.71–0.93)	< 0.01
3.Serum 25(OH)D 50.1–75.0 nmol/l	0.90 (0.79–1.04)	0.15
**Model 3:** *Model 2 + ethnicity*		
1.Serum 25(OH)D ≤ 25.0 nmol/l	0.75 (0.62–0.89)	< 0.01
2.Serum 25(OH)D 25.1–50.0 nmol/l	0.84 (0.73–0.97)	0.02
3.Serum 25(OH)D 50.1–75.0 nmol/l	0.91 (0.79–1.05)	0.20
**Model 4:** *Model 3 + W/H ratio*, *Chol/HDL ratio*		
1.Serum 25(OH)D ≤ 25.0 nmol/l	0.76 (0.65–0.91)	< 0.01
2.Serum 25(OH)D 25.1–50.0 nmol/l	0.84 (0.73–0.96)	0.01
3.Serum 25(OH)D 50.1–75.0 nmol/	0.91 (0.80–1.04)	0.19
**Model 5:** *Model 4 + season*		
1.Serum 25(OH)D ≤ 25.0 nmol/l	0.76 (0.63–0.91)	< 0.01
2.Serum 25(OH)D 25.1–50.0 nmol/l	0.83 (0.72–0.96)	0.01
3.Serum 25(OH)D 50.1–75.0 nmol/l	0.91 (0.79–1.04)	0.16

BMI, body mass index; Chol/HDL ratio, cholesterol–high-density lipoprotein ratio; W/H ratio, waist to hip ratio

* Vitamin D group with serum 25(OH)D > 75 nmol/l is the reference group

**HOMA-IR natural logarithm (0.76 means 1–0.76 = 24% higher HOMA-IR in vitamin D group ≤ 25 nmol/l than > 75 nmol/l)

### Lipid profile

Mean total cholesterol (TC) /HDL ratio was 3.9 ± 1.4 mmol/L in our study population. The ratio differed significantly between the vitamin D groups with a difference of -0.79 mmol/L between the lowest and highest vitamin D group (p < 0.01). After correcting for confounders, the association did not remain significant between the lowest and highest vitamin D groups (B = -0.31; 95% CI: -0.73–0.12, p = 0.16). A significant difference was seen in HDL-cholesterol between the lowest and highest vitamin D group after correction for confounders (B = 0.20; 95% CI: 0.05–0.60, p < 0.01). Apolipoprotein A1 was significantly higher in the highest vitamin D group compared to the lowest group after correction for confounders (B = 26.2; 95% CI: 7.5–45.0, p < 0.01). A small significant difference was seen in serum total cholesterol demonstrating higher serum total cholesterol in the highest vitamin D group compared to the lowest vitamin D group (B = 0.41; 95% CI: 0.02–0.70, p = 0.05). No difference between the vitamin D groups was found in serum triglycerides, LDL-cholesterol, and apolipoprotein B (Table 5).

**Table 5:** Regression analysis of serum 25(OH)D > 75 nmol/l versus < 25 nmol/l and parameters of lipid profile.

	Model I		Model II		Model III	
	B (95% CI)	p	B (95% CI)	p	B (95% CI)	p
TC (mmol/L)	0.16 (-0.13–0.46)	0.27	0.34 (0.04–0.65)	0.03	0.41 (0.02–0.70)	0.05
TG (mmol/L)	-0.28 (-0.44 –-0.12)	< 0.01	-0.12 (-0.28–0.04)	0.13	-0.14 (-0.35–0.06)	0.18
HDL (mmol/L)	0.34 (0.23–0.45)	< 0.01	0.23 (0.12–0.34)	< 0.01	0.20 (0.05–0.34)	< 0.01
LDL (mmol/L)	0.05 (-0.20–0.29)	0.70	0.24 (0.00–0.49)	0.06	0.28 (-0.04–0.60)	0.09
TC/HDL ratio (mmol/L)	-0.79 (-1.12 –-0.46)	< 0.01	-0.33 (-0.65 –-0.01)	0.048	-0.31 (-0.73–0.12)	0.16
Apo A1 (g/L)	23.1 (9.2–37.0)	< 0.01	18.6 (4.1–33.1)	0.01	26.2 (7.5–45.0)	< 0.01
Apo B (g/L)	-9.8 (-18.9 –-0.8)	0.03	-0.03 (-9.1–9.0)	0.99	7.9 (-3.6–19.5)	0.18

Model 1: crude.

Model 2: adjusted for BMI, fasting glucose

Model 3: adjusted for BMI, fasting glucose, season, ethnicity and age

25(OH) D, 25-hydroxy vitamin D; Apo, apolipoprotein; HDL, high density lipoprotein; LDL, low density lipoprotein; TC, total cholesterol; TG, triglycerides

## Conclusions

The aim of this study was 1) to explore vitamin D status in women with PCOS compared to fertile controls and 2) to evaluate the associations between vitamin D status and metabolic disturbances in PCOS women. In this study the lower vitamin D status in women with PCOS compared to fertile controls was confirmed [20]. Low serum 25(OH)D status is significantly associated with a higher insulin resistance in women with PCOS independent of major confounders as BMI, season and ethnicity. Moreover, women with PCOS and a severe vitamin D deficiency had the lowest levels of HDL cholesterol and apolipoprotein A1.

The strength of our study is that we have studied the largest cohort of PCOS women and compared our findings with a comparison group without PCOS, which allows a well-powered comparison. Second, all samples were measured using the LC-MS/MS method, which is the golden standard for measurement of serum 25(OH)D [27].

A disadvantage of our study is that metabolic measurements were not available in the fertile control women. Although the control women were enrolled from the general population and not selected on fertility, it is not likely that a substantial part of the control women had PCOS due to the fact that they all had a spontaneous pregnancy and regular menstrual cycles before pregnancy. The blood collection of the control women was carried out 15 to 17 months after delivery to exclude the effect of pregnancy on vitamin D metabolism. Another limitation of our study could be that the comparison group was not originally created as a control group for this study and was matched retrospectively, resulting in a small difference in age and BMI between both groups. Taken this into account we labelled the study not as a case-control study, but as a comparison study. However, this was the best control group available in the same region and age category, of which we were certain all women had a spontaneous pregnancy which makes the chance of PCOS and thus confounding in this group very low.

Although serum 25(OH)D is stable over time, theoretically long-time storage of serum samples at -80 ⁰C could have influenced the vitamin D concentration [29]. However, any influence of the storage time on vitamin D level does not seem very likely as the mean storage time in control women, which demonstrated to have a higher mean serum 25(OH)D concentration, was significantly higher than in PCOS women (10 versus 6 years).

Another important point is the possibility of residual confounding which cannot be ruled out, despite correction for several confounders. Last limitation could be that we have used a surrogate marker of insulin resistance (HOMA-IR) and not the golden standard to measure insulin resistance, i.e. insulin clamp technique.

### Interpretation of study findings

Several epidemiologic studies have explored the effect between vitamin D status and metabolic disturbances in PCOS women and controls. These studies are summarized in an earlier systematic review [20], in which was concluded that serum 25(OH)D is a significant predictor for insulin resistance in PCOS women. However, this effect disappeared after adjustment for BMI. Important limitations of the earlier performed observational studies were the small sample sizes and poor analyses with lack of correcting for confounding factors as ethnicity, season of blood collection and BMI.

The finding of a higher prevalence of vitamin D defiency in PCOS women compared to (fertile) controls is in line with earlier reports, which observed a percentage of vitamin D deficiency (defined as serum 25(OH)D < 50nmol/l) of 67–85% among women with PCOS [19].

The mean vitamin D level in control women of our study was comparable with the overall mean vitamin D level that was reported in control women in the earlier performed systematic review [20]. However, the mean vitamin D level in women with PCOS appeared to be much lower than reported in the same review. This discrepancy could be explained by the finding of a discrepant higher vitamin D level in PCOS women compared to controls in some of the reviewed studies, which could be caused by the lack of adjustment for major confounders (i.e. the season of measurement of vitamin D level and ethnicity) [30–32]. Moreover, PCOS women with hirsutism could have less sun exposure and consequently a lower vitamin D synthesis.

To date, there is still a debate what the optimal serum 25(OH)D level is. The Institute of Medicine [33] has determined that serum 25(OH)D levels greater than 50 nmol/l are sufficient based on the current studies available, although other experts advise that optimal levels should be higher (> 75 nmol/l) [34]. In our study population only 138/639 (22%) had a serum 25(OH)D concentration > 75 nmol/l. In the groups with lower serum 25(OH)D a more disturbed metabolic profile was seen; higher levels of insulin resistance and a worse lipid profile, supporting the recommendation from the Endocrine Society to reach a serum 25(OH)D concentration > 75 nmol/l.

Vitamin D is thought to influence the development of PCOS through gene transcription and influences metabolism [35]. The effect of vitamin D status on glucose metabolism appears to be mediated by direct and indirect pathways. A direct effect on insulin secretion may be mediated by activation of VDRs in the pancreatic beta-cell with the addition of the presence of 1α-hydroxylase to locally produce 1,25(OH)_2_D. Furthermore, the direct effect of vitamin D on insulin secretion is supported by the presence of the vitamin D-response element in the human insulin promoter gene [36]. Vitamin D deficiency may also increase systemic inflammation, known to play an important role in the pathogenesis of insulin resistance [18]. Finally, insulin secretion and insulin resistance are both calcium-dependent processes. Both could be influenced by vitamin D status through an alteration in calcium concentration and flux through cell membranes of pancreas and insulin-responsive tissues.

Next to the linkage of vitamin D to the pathogenesis of PCOS, vitamin D may also effect fertility outcomes in PCOS [37]. Vitamin D supplementation may improve ovulatory dysfunction and thereby fertility in PCOS women [38, 39]. This may be explained by the finding that VDR has been found across several tissues within the female reproductive system (i.e. ovarian, decidua, placenta and endometrium cells) [40–41]. Furthermore, 1,25(OH)D_2_ directly leads to the production of estrogen and progesterone in cultured human ovary and placental cells [42,43]. Moreover, an inverse association between vitamin D concentration and anti-Müllerian hormone (AMH) has been reported. Elevated AMH reflects abnormal folliculogenesis in PCOS women [44,45]. In the literature women more often have lower vitamin D levels compared to men [46]. An underlying association between vitamin D levels with androgens as an explanation could, however, not be found[38].

The association found between vitamin D and dyslipidemia (i.e. HDL-cholesterol and apolipoprotein A1) had previously been described [47]. Low HDL-cholesterol is one of the central features of metabolic syndrome. As dyslipidemia should be considered as an additional therapeutic target in women suffering from PCOS [48], vitamin D might be useful in the complex treatment of these women.

Apart from these cross-sectional findings, several intervention trials examined the effects of vitamin D supplementation on metabolic parameters and clinical parameters including fertility in PCOS women. These studies differed in study design, population, dosing regimens, as well as study outcomes [40,49]. A significant inverse association was found between serum 25(OH)D and insulin resistance in the included observational studies, which is in line with our results. Some RCTs had promising results regarding vitamin D effects on metabolic as well as on fertility aspects. However, overall no significant improvement in metabolic functions and fertility outcome was found among PCOS women supplemented with vitamin D.

This could be due to the inconsistencies in study design, included PCOS populations and duration of follow up. The largest study with a RCT design among 104 obese, vitamin D deficient PCOS women did reveal a positive effect of weekly 50.000 IU vitamin D plus calcium 1000mg/day on insulin resistance [50].

In conclusion, this is the largest epidemiologic study showing a significant association between vitamin D status and metabolic disturbances in patients with PCOS. Moreover, PCOS women had a significant lower serum 25(OH)D compared to fertile controls. For future research large randomized controlled clinical trials are necessary to explore whether this association has a causal linkage.

## Supporting information

S1 Data Data_case_control_PLOS. (SAV)
